# Supplementary material for: Facile production of ultra-fine silicon nanoparticles
Source: R Soc Open Sci. 2020 Sep 16;7(9):200736. doi: 10.1098/rsos.200736 (PMC7540795; doi:10.1098/rsos.200736)
Supplement: Supplementart data [file rsos200736supp1.docx]

**SUPPLEMENTARY INFORMATION**

Facile production of ultra-fine silicon nanoparticles

Klaudia Tokarska, Qitao Shi, Lukasz Otulakowski, Pawel Wrobel, Huy Quang Ta, Przemyslaw Kurtyka, Aleksandra Kordyka, Mariola Siwy, Margaryta Vasylieva, Aleksander Forys, Barbara Trzebicka, Alicja Bachmatiuk, Mark H. Rümmeli*

^1^ Centre of Polymer and Carbon Materials Polish Academy of Sciences (CMPW PAN), M. Curie-Sklodowskiej 34, Zabrze 41-819, Poland

^2^ Soochow Institute for Energy and Materials Innovations (SIEMIS), College of Energy, Key Laboratory of Advanced Carbon Materials and Wearable Energy Technologies of Jiangsu Province, Soochow University, Suzhou 215006, China

^3^ The Leibniz Institute for Solid State and Materials Research Dresden (IFW Dresden), Institute for Complex Materials, Helmholtzstrasse 20, 01069 Dresden, Germany

^4^ Department of Biomaterials and Medical Devices Engineering, Faculty of Biomedical Engineering, Silesian University of Technology, Roo-sevelta 40, Zabrze 41-800, Poland

^5^ Institute of Environmental Technology, VSB-Technical University of Ostrava, 17. Listopadu 15, Ostrava 708 33, Czech Republic

^*^ Corresponding author (email: mhr1967@yahoo.com)

**Abstract**

A facile procedure for the synthesis of ultra-fine silicon nanoparticles without the need for a Schlenk vacuum line is presented. The process consists of the production of a (HSiO_1.5_)_n_ sol-gel precursor based on the polycondensation of low-cost trichlorosilane (HSiCl_3_), followed by its annealing and etching. The obtained materials were thoroughly characterized after each preparation step by electron microscopy, Fourier transform and Raman spectroscopy, x-ray dispersion spectroscopy, diffraction methods and photoluminescence spectroscopy. The data confirms the formation of ultra-fine silicon nanoparticles with controllable average diameters between 1 and 5 nm depending on the etching time.


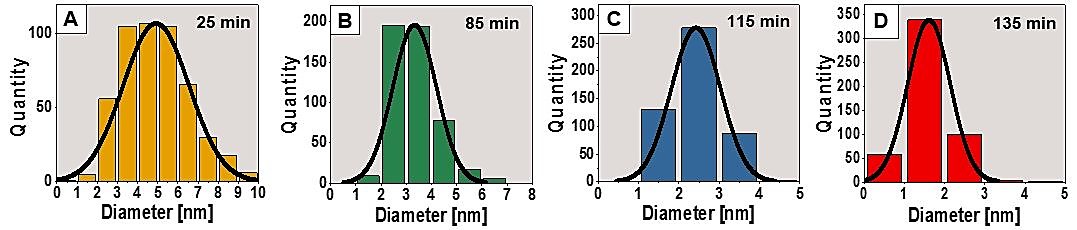


Figure S1. Decreasing the size of silicon nanoparticles with longer etching times. A particle size distribution histograms of silicon nanoparticles after the third final preparation step after (A) 25 min, (B) 85 min, (C) 115 min and (D) 135 min of etching.


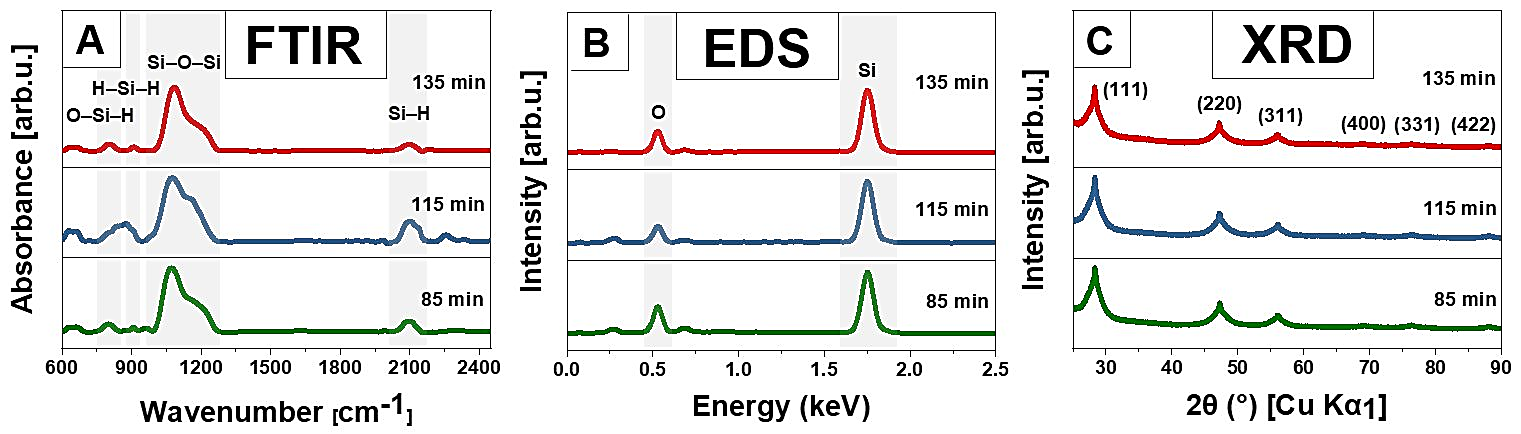


Figure S2. Confirmation of the crystallinity of ultrafine Si nanoparticles after different etching times: (A) Fourier transform infrared (FTIR) spectrum, (B) energy-dispersive X-ray spectroscopy (EDS) and (C) X-ray powder diffraction (XRD) of SiNP after 85 min, 115 min and 135 min of etching
